# HAPLN1 Affects Cell Viability and Promotes the Pro-Inflammatory Phenotype of Fibroblast-Like Synoviocytes

**DOI:** 10.3389/fimmu.2022.888612

**Published:** 2022-06-02

**Authors:** Yong Chen, Baojiang Wang, Yanjuan Chen, Qunyan Wu, Wing-Fu Lai, Laiyou Wei, Kutty Selva Nandakumar, Dongzhou Liu

**Affiliations:** ^1^ Division of Rheumatology and Research, The Second Clinical Medical College, Jinan University (Shenzhen People’s Hospital), Shenzhen, China; ^2^ Institute of Maternal and Child Medicine, Affiliated Shenzhen Maternity and Child Healthcare Hospital, Southern Medical University, Shenzhen, China; ^3^ School of Basic Medicine, Jinan University, Guangzhou, China; ^4^ Department of Urology, Zhejiang Provincial People’s Hospital, Hangzhou Medical College, Zhejiang, China; ^5^ Department of Applied Biology and Chemical Technology, Hong Kong Polytechnic University, Wanchai, Hong Kong SAR, China; ^6^ Southern Medical Universit - Karolinska Institute (SMU-KI) United Medical Inflammation Center, School of Pharmaceutical Sciences, Southern Medical University, Guangzhou, China

**Keywords:** rheumatoid arthritis, hyaluronan and proteoglycan link protein 1, fibroblast-like synoviocytes, cell viability, cell migration, pathogenesis

## Abstract

HAPLN1 maintains aggregation and the binding activity of extracellular matrix (ECM) molecules (such as hyaluronic acid and proteoglycan) to stabilize the macromolecular structure of the ECM. An increase in HAPLN1 expression is observed in a few types of musculoskeletal diseases including rheumatoid arthritis (RA); however, its functions are obscure. This study examined the role of HAPLN1 in determining the viability, proliferation, mobility, and pro-inflammatory phenotype of RA- fibroblast-like synoviocytes (RA-FLSs) by using small interfering RNA (siHAPLN1), over-expression vector (HAPLN1^OE^), and a recombinant HAPLN1 (rHAPLN1) protein. HAPLN1 was found to promote proliferation but inhibit RA-FLS migration. Metformin, an AMPK activator, was previously found by us to be able to inhibit FLS activation but promote HAPLN1 secretion. In this study, we confirmed the up-regulation of HAPLN1 in RA patients, and found the positive relationship between HAPLN1 expression and the AMPK level. Treatment with either si-HAPLN1 or HAPLN1^OE^ down-regulated the expression of AMPK-ɑ gene, although up-regulation of the level of p-AMPK-ɑ was observed in RA-FLSs. si-HAPLN1 down-regulated the expression of proinflammatory factors like TNF-ɑ, MMPs, and IL-6, while HAPLN1^OE^ up-regulated their levels. qPCR assay indicated that the levels of TGF-β, ACAN, fibronectin, collagen II, and Ki-67 were down-regulated upon si-HAPLN1 treatment, while HAPLN1^OE^ treatment led to up-regulation of ACAN and Ki-67 and down-regulation of cyclin-D1. Proteomics of si-HAPLN1, rHAPLN1, and mRNA-Seq analysis of rHAPLN1 confirmed the functions of HAPLN1 in the activation of inflammation, proliferation, cell adhesion, and strengthening of ECM functions. Our results for the first time demonstrate the function of HAPLN1 in promoting the proliferation and pro-inflammatory phenotype of RA-FLSs, thereby contributing to RA pathogenesis. Future in-depth studies are required for better understanding the role of HAPLN1 in RA.

## 1 Introduction

Rheumatoid arthritis (RA) is a chronic autoimmune disease characterized by destructive, chronic, debilitating arthritis. Pannus formation is one of the main features of the disease (1). During RA development, the number of fibroblast-like synoviocytes (FLSs), one of the key effector cells (2), increases significantly, leading to the transformation of the synovial lining from a delicate structure into an invasive hyperplastic tissue mass known as pannus (3). The pannus tissue contributes to the chronic inflammatory milieu in the arthritic joint, resulting in an expansion of the synovial membrane and the occurrence of a complex interplay among different dendritic cell (DC) subtypes, T cells, macrophages, B cells, neutrophils, fibroblasts, and osteoclasts (4). FLSs transform the synovium into a hyperplastic invasive pannus by producing various mediators such as cytokines and proteases, leading to the destruction of the extracellular matrix (ECM), cartilages, and bones (5). The cell phenotype of RA-FLSs mimics cancer cells in terms of invasion, proliferation, and apoptosis (6). The presence of functionally distinct arthritis-associated fibroblast subsets in human synovial tissues, along with the identification of fibroblast subsets that mediate either inflammation or bone/cartilage damage in arthritis, suggests that targeting FLSs is an attractive therapeutic approach to treat RA (7).

Hyaluronan and proteoglycan link protein 1 (HAPLN1), also known as cartilage link protein, was originally identified from the proteoglycan component extracted from the bovine articular cartilage (8). Our previous study confirmed the secretion of HAPLN1by RA-FLSs (9). Physiologically, HAPLN1 is a component of the ECM required for normal cartilage development. It maintains stable aggregation and the binding activity of two important ECM macromolecules [hyaluronic acid (HA) and proteoglycan] which, along with other molecules present in the joint, contribute not only to the maintenance of the stable macromolecular structure but also to the compression resistance of the joint (10). The vital role played by HAPLN1 in regulating bone/cartilage growth was documented previously. HAPLN1-deficient mice showed a series of cardiac malformations (e.g., atrial septal and myocardial defects) along with a remarkable reduction in the level of multifunctional proteoglycans (11). The most classic disease first reported to be associated with HAPLN1 is juvenile rheumatoid arthritis (12), but the mechanisms were not yet fully known. Nevertheless, genomics research has enabled the clarification of the relation of HAPLN1 with various rheumatic disorders [including spinal degeneration, osteoarthritis (OA), ankylosing spondylitis (AS), and RA] (13–17). The HAPLN1 gene is present adjacent to the ankylosing spondylitis-associated single nucleotide polymorphism (SNP) (rs4552569) (18). Compared to subjects with the CC or CT genotype, those with the TT genotype for the SNP at rs179851 were found to be significantly overrepresented among the subjects with higher scores for osteophyte formation and disc space narrowing (13). Importantly, HAPLN1 showed a significant correlation with the severity of RA (17). We found that one of the most significantly up-regulated genes in RA-FLSs, contrary to osteoarthritis (OA)-FLSs, is HAPLN1, whose protein level increases in the plasma and synovium of RA patients (9). Recently, the oncogenic phenotype of HAPLN1 was reported to be able to contribute to cellular hyperactivity and remodeling of collagen matrices (19, 20). The exact role of HAPLN1 in RA and its interaction with other matrix molecules under disease conditions are still unclear. The objective of this study is, therefore, to explore the potential role played by HAPLN1 in RA-FLS-mediated disease pathogenesis.

## 2 Results

### 2.1 Increased HAPLN1 Expression in the Synovium and Plasma of RA Patients

**Figure 1:**
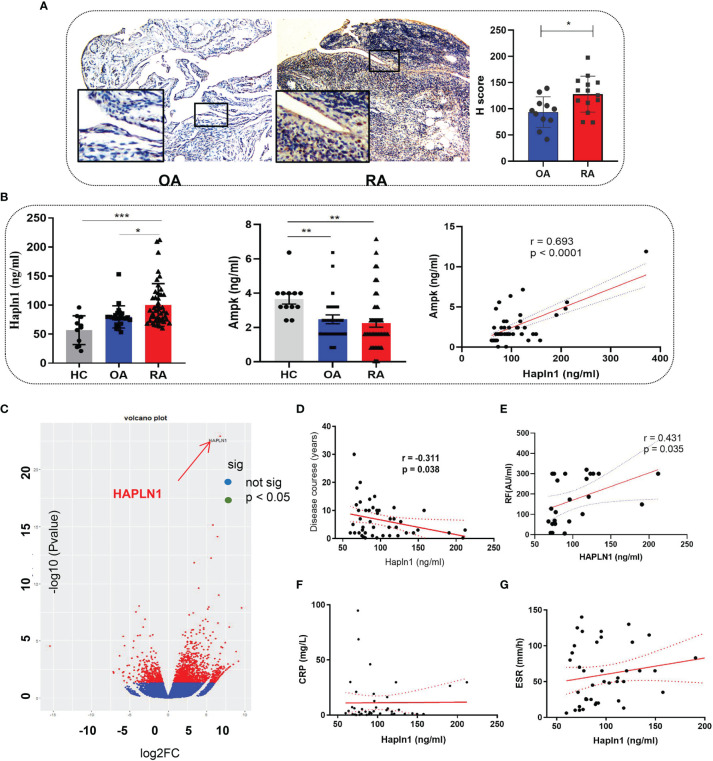
HAPLN1 is up-regulated in RA patients and positively correlated with AMPK. **(A)** Increased HAPLN1 expression, quantified by the H score, in RA (n=20) than OA (n=17) synovium (magnification of gross looking, 10×5; magnification of foci in the framed, 10×20). **(B)** Plasma HAPLN1 levels examined by ELISA were significantly enhanced in RA (n=61) patients than OA (n=20) patients and HC (n=12), while AMPK levels were decreased in both OA and RA patients compared to HC. Plasma HAPLN1 and AMPK levels were significantly positively correlated (r = 0.693, p < 0.0001). **(C)** Volcano plot showing higher expression of the HAPLN1 gene in FLSs from RA than OA patients (n=3 in each group). **(D, E)** Plasma HAPLN1 levels negatively correlated with the disease course (r = -0.311, p = 0.038) and showed a moderately positive correlation with RF levels (r = 0.431, p = 0.038) of RA patients. **(F, G)** No significant correlation between the HAPLN1 level and ESR and CRP. *p < 0.05; **P < 0.01; ***p < 0.001.

The HAPLN1 expression in the synovium of RA (n=20) was found by immunohistochemical assays to be significantly higher than that of OA patients (n=17) (**Figure 1A** and **Supplementary Table S1** for participants’ details). Similarly, the HAPLN1 level in the plasma of RA patients (n=61) was higher than that of OA patients (n=20) and healthy control subjects (HC, n=12) (**Figure 1B** and **Supplementary Table S2** for participants’ details). These results agree with our earlier mRNA sequencing analysis, which showed that the level of HAPLN1 expression in RA-FLSs is higher than that in OA FLSs (**Figure 1C**) (9).

The AMP-activated protein kinase (AMPK) pathway contributes to cell viability, metabolism, and inflammation during the onset and progression of RA (21, 22). Treatment of RA-FLSs with an AMPK activator, metformin, up-regulates HAPLN1 expression (9). Therefore, changes in AMPK expression could help elucidate the functions of HAPLN1. In this study, the AMPK level in healthy people was found to be significantly higher than OA and RA patients and had a significant positive correlation with the HAPLN1 level in the plasma of RA patients (n = 48; r = 0.693, p < 0.0001) (**Figure 1C**). In addition, the plasma HAPLN1 level negatively correlated with the course of the arthritis disease (n = 46, r = -0.311, p = 0.038). HAPLN1 in RA patients having less than 3 years of disease activity (n = 20) was higher than that in patients having disease symptoms for more than 3 years (n = 41) (**Figure 1D** and **Supplementary Figure S1**). A moderate positive correlation between HAPLN1 and the rheumatoid factor was noted (n = 24, r = 0.431, p < 0.05) (**Figure 1E**). However, no correlation between the HAPLN1 level and other indexes of disease activity (such as ESR and CRP) was revealed by the data in this study (**Figures 1F, G**). The elevated AMPK level was consistent with our previous observations (9). It is plausible that HAPLN1 participates in AMPK-regulated metabolic pathways.

### 2.2 HAPLN1 Increased the Proliferation But Inhibited the Mobility of RA-FLSs

To dissect the role of HAPLN1 in determining the viability of RA-FLSs, three small interfering RNA (si-RNA HAPLN1) molecules and an over-expression plasmid vector (HAPLN1^OE^) were used to study the proliferation and migration ability of RA-FLSs. All of the tested siRNA molecules in this study effectively down-regulated the mRNA level of HAPLN1, while transfecting RA-FLSs with HAPLN1^OE^ up-regulated the expression of HAPLN1 (**Supplementary Figure S2**). Recombinant HAPLN1 (rHAPLN1) at different concentrations was also added to RA-FLSs to study its functions.

**Figure 2:**
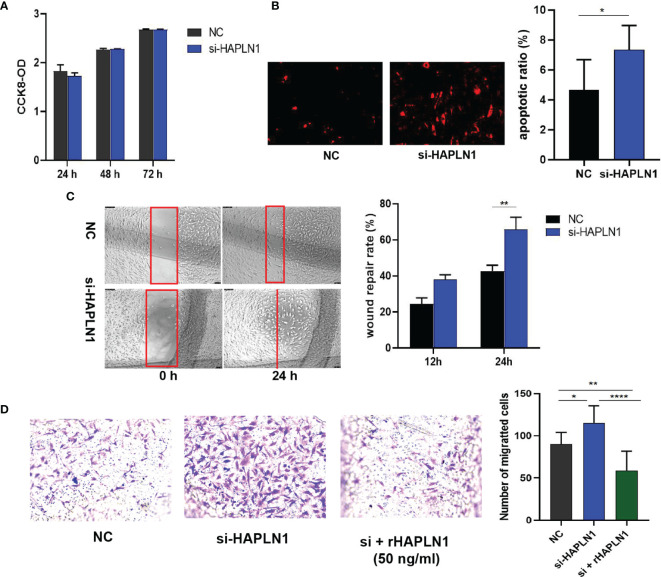
Effects of si-HAPLN1 on RA-FLS activity. Transfection of siHAPLN1 in RA-FLSs did not significantly affect the **(A)** proliferation but significantly increased **(B)** apoptosis (magnification: 10×10) and **(C, D)** migration ability of RA-FLSs. Wound healing **(C**, magnification: 10×5**)** and transwell assays **(D**, magnification: 10×5**)** were used to measure the migration capacity of FLSs. Recombinant HAPLN1 (50 ng/ml) attenuated the increased migration ability induced by si-HAPLN1 in the transwell assay. *p < 0.05; **p < 0.01; ****p < 0.0001. NC, negative control is control siRNA of si-HAPLN1.

**Figure 3:**
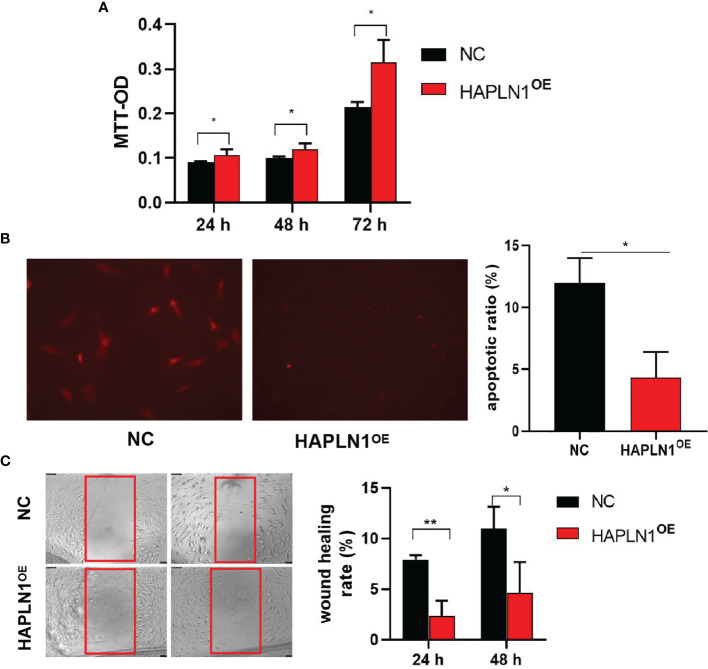
Effects of HAPLN1 over-expression on RA-FLSs activity. Over-expression of HAPLN1 (HAPLN1^OE^) in RA-FLSs significantly increased the **(A)** proliferation but reduced **(B)** apoptosis (magnification: 10×10) and **(C)** migration (magnification: 10×5) of RA-FLSs. *p < 0.05; **p < 0.01. NC, negative control is control plasmid vector of HAPLN1^OE^.

**Figure 4:**
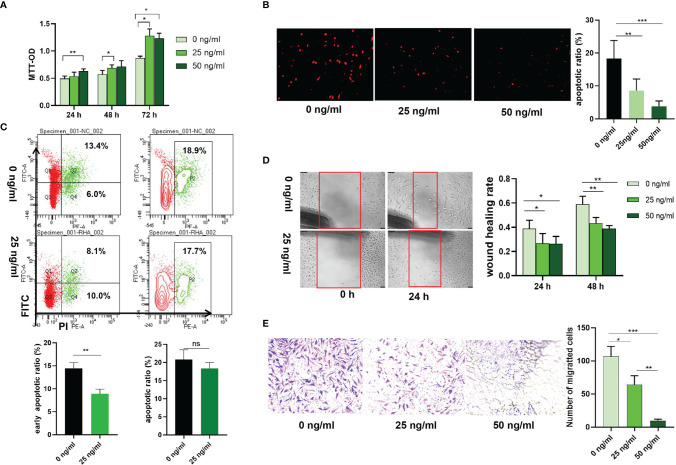
Effects of rHAPLN1 on RA-FLSs activity. **(A)** Treatment of RA-FLSs with rHAPLN1 significantly enhanced the **(A)** proliferation but reduced **(B, C)** apoptosis of RA-FLSs (magnification: 10×10), especially during the early phase and **(D, E)** migration. Wound healing **(D)** and transwell **(E)** assays were used to evaluate the migration capacity of FLSs (magnification:10×5). ns, not significant; *p < 0.05; **p < 0.01; ***p < 0.001.

Transfection with si-HAPLN1 did not affect the proliferation of RA-FLSs (**Figure 2A** and **Supplementary Figure S3**), though a significant increase in the apoptotic ratio of RA-FLSs was revealed by the results of our TUNEL assay (**Figure 2B**). The wound healing assay and the transwell assay showed an increase in the migration ability of RA-FLSs after transfection with si-HAPLN1 (**Figures 2C, D**), and the effect was reversed after the addition of rHAPLN1 (50 ng/ml) (**Figure 2D**). Conversely, after transfection with HAPLN1^OE^, RA-FLSs showed enhanced proliferation and a decline in the apoptotic ratio (**Figures 3A, B** and **Supplementary Figure S4**). Results of the wound healing assay showed a decrease in the level of migration exhibited by HAPLN1^OE^-transfected RA-FLSs (**Figure 3C**), whereas rHAPLN1 confirmed the effects of HAPLN1 on RA-FLSs which showed a significant increase in the proliferation activity (**Figure 4A** and **Supplementary Figure S5**) and a reduction in apoptosis, especially during the early phase (**Figures 4B, C**). The wound healing assay and the transwell assay demonstrated that rHAPLN1 inhibited the migration ability of RA-FLSs (**Figures 4D, E**). It is known that cell mobility is elevated in cancer cells as well as in activated RA-FLSs (6). However, our experiments with si-HAPLN1, HAPLN1^OE^, and rHAPLN1 demonstrated that HAPLN1 shows an inhibitory effect on the mobility of RA-FLSs although it could activate RA-FLS proliferation.

### 2.3 Expression of HAPLN1 Affects the Cell Cycle, Structure Molecules and Cytokines Secretion of RA-FLSs

#### 2.3.1 The mRNA Level of Ki-67 Was Up-Related by HAPLN1

**Figure 5:**
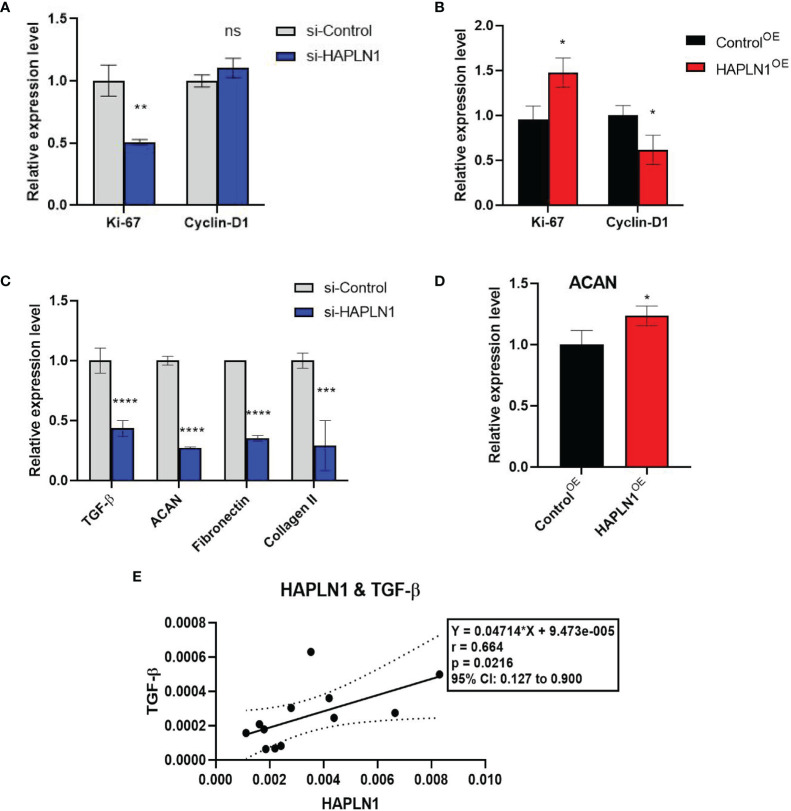
The effects of si-HAPLN1 and HAPLN1^OE^ on RA-FLS-derived cell-cycle and structure molecules. **(A)** si-HAPLN1 transfection decreased Ki-67 mRNA expression significantly, **(B)** HAPLN1^OE^ transfection increased Ki-67 but down-regulated Cyclin D1 expression in RA-FLSs. **(C)** Expression of mRNA of TGF-β, ACAN, fibronectin, and collagen II were down-regulated by si-HAPLN1 treatment in RA-FLSs, while **(D)** ACAN was significantly up-regulated by HAPLN1^OE^ transfection. **(E)** Pearson correlation coefficient analysis showed relative mRNA levels from the si-control group, with a strong positive correlation (r = 0.66, 95%CI [0.13, 0.90], *p* < 0.05) between TGF-β and HAPLN1. *p < 0.05; **p < 0.01; ***p < 0.001; ****p < 0.0001; ns, not significant.

**Figure 6:**
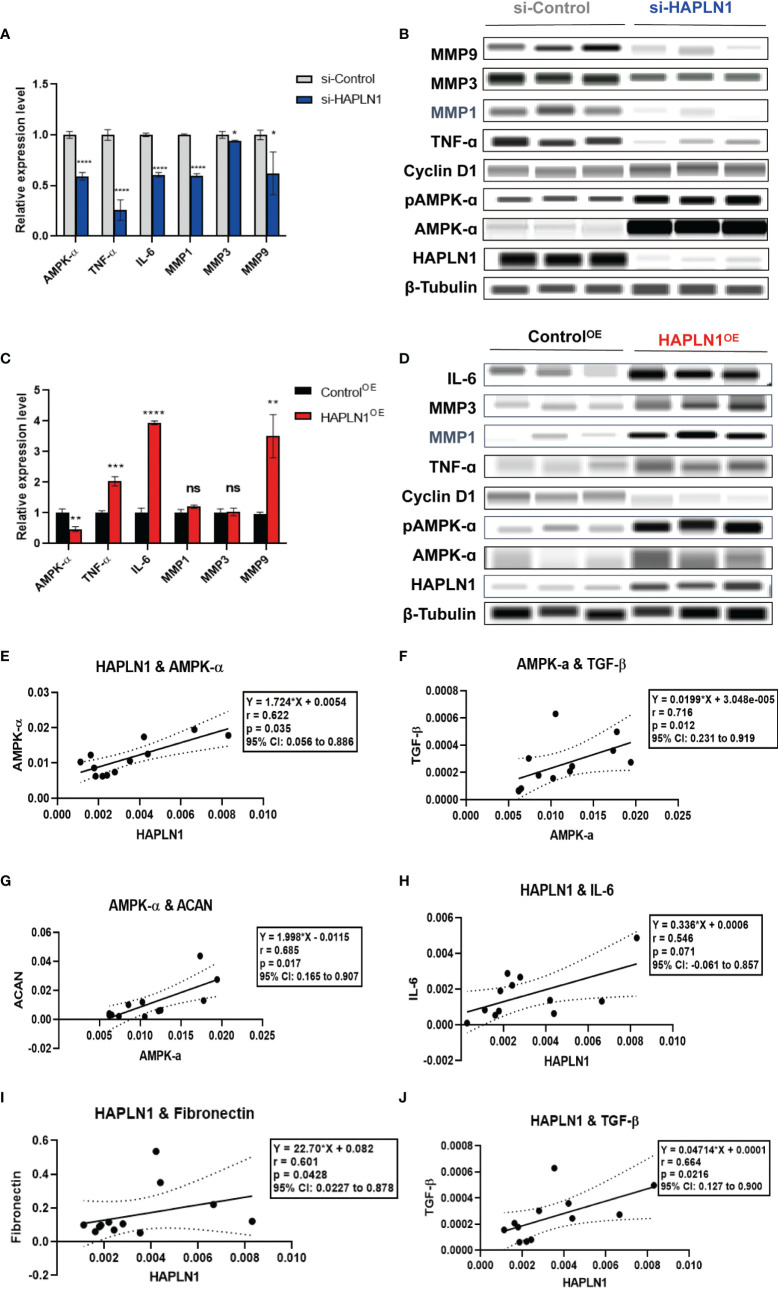
The effects of si-HAPLN1 and HAPLN1^OE^ on RA-FLS-derived cytokines. **(A, B)** si-HAPLN1 transfection to RA-FLSs inhibited TNF-ɑ, IL-6, MMP1, MMP3, and MMP9 mRNA and protein expression; the mRNA level of AMPK-ɑ was reduced, but AMPK-ɑ and pAMPK-ɑ were up-regulated in the protein level. Expression of Cyclin D1 seems unaffected by si-HAPLN1 transfection. **(C, D)** After over-expression of HAPLN1 in RA-FLSs, TNF-ɑ, IL-6, and MMP9 mRNA and protein expressions were up-regulated while AMPK-ɑ was down-regulated at the mRNA level but AMPK-ɑ and pAMPK-ɑ were up-regulated at the protein level. Cyclin D1 proved to be down-regulated in the protein level by HAPLN1^OE^ transfection. **(E)** In control si-RNA-treated RA-FLSs form multiple tests, **(E–G)** AMPK-ɑ mRNA positively correlated with HAPLN1, TGF-β, and ACAN. **(H-J)** HAPLN1 mRNA shows positive correlations with fibronectin, TGF-β, and IL-6 expression. *p < 0.05; **p < 0.01; ***p < 0.001; ****p < 0.0001; ns, not significant. si-Control, negative control management of si-HAPLN1 transfection; Control^OE^, negative control management of HAPLN1^OE^ transfection.

In cancer research, Ki-67 expression can indicate the effect of a treatment on cell proliferation (23), and is proved to be able to predict the activity of RA-FLSs (24). Transfection with si-HAPLN1 decreased Ki-67 mRNA expression significantly, while transfection with HAPLN1^OE^ increased its expression in RA-FLSs (**Figures 5A, B**). We also checked the expression of Cyclin D1, which is an important regulator of cell cycle, with many carcinomas being characterized by Cyclin D1 overexpression that induces uncontrolled cell proliferation (25). Changes of Cyclin D1 are not consistent with the changes observed in the MTT assay, CCK8 assay, and Ki-67 expression in the current study. It was significantly reduced by HAPLN1^OE^ transfection (**Figures 5A, B**, **6C, D**). It seems Cyclin D1 could not validate HAPLN1 promotes cell viability, but inhibits cell mobility function could be explained as Cyclin D1 is reported to target roles in cytoskeletal modelling, cell adhesion, and motility. Cyclin D1 loss is associated with reduced cellular migration in response to different stimuli (25).

#### 2.3.2 Cell Structure Molecules Were Up-Regulated by HAPLN1

ACAN is a well-documented chaperone of HAPLN1, playing a significant role in regulating the ECM structure (26). mRNA of ACAN was significantly down-regulated by si-HAPLN1 while up-regulated by HAPLN1^OE^ transfection. Other structure molecules such as TGF-β, fibronectin, and collagen II were also down-regulated by si-HAPLN1 treatment (**Figures 5C, D**). These targets play key roles in ECM formation, wound healing, and fibrosis in different pathological conditions. A strengthened ECM architecture is a way to restrain cancer cell progression (27). Thus, changes in these targets as led by HAPLN1 validated the role of HAPLN1 in controlling cell migration of RA-FLSs. Pearson correlation coefficient analysis was applied to analyze the relative mRNA levels from the si-control group. A strong positive correlation (r = 0.66, 95%CI [0.13, 0.90], p < 0.05) between TGF-β and HAPLN1 was observed (**Figure 5E**). TGF-β signaling events are known to control diverse processes and responses, such as cell proliferation, differentiation, apoptosis, and migration. Besides limited cell migration in pre-malignant cells (28), it cross-talks with multiple inflammation pathways (29).

#### 2.3.3 Inflammatory Cytokines Were Promoted by HAPLN1

Previously we reported an increase in the expression of HAPLN1 in RA-FLSs after stimulation of the AMPK pathway (9). We further confirmed the inter-dependence of the positive correlation between HAPLN1 and AMPK by using clinical RA plasma samples as mentioned above (**Figure 1B**). The role of AMPK as a regulator of metabolism and inflammation is well known (30, 31). Therefore, we investigated how HAPLN1 affects RA-FLSs in the expression of AMPK-ɑ and related cytokines, such as TNF-ɑ, IL-6, and MMPs, involved in inflammation.

Transfection of si-HAPLN1 into RA-FLSs inhibited the expression of mRNAs of AMPK-ɑ, TNF-ɑ, IL-6, and MMP1, MMP3, and MMP9 (**Figure 6A**). Results of the automated WB assay confirmed successful silencing of HAPLN1. The expression of TNF-ɑ, MMP1, MMP3, and MMP9 was inhibited in a way similar to the expression of the corresponding mRNAs (**Figure 6B**). However, the expression of AMPK-ɑ and pAMPK-ɑ at the protein level, unlike its mRNA expression, was found to be up-regulated. HAPLN1^OE^ transfection in RA-FLSs up-regulated the expression of TNF-ɑ, IL-6, and MMP9, while AMPK-ɑ was down-regulated (**Figure 6C**). Unlike the expression of mRNA, the expression of AMPK-ɑ and pAMPK-ɑ was also up-regulated when TNF-ɑ, IL-6, MMP1, and MMP3 showed a trend of up-regulation (**Figure 6D**).

To better understand the potential interactions between these molecules, we collected multiple expression data of relative mRNA levels from control groups of si-HAPLN1 (si-Control), and applied the Pearson correlation coefficient analysis. HAPLN1 levels showed a strong positive correlation with the level of AMPK-ɑ (**Figure 6E**), which is in accordance with its plasma levels and also with our previous findings using metformin treatment (9). AMPK-ɑ also positively correlated to TGF-β and ACAN (**Figures 6F, G**). Importantly, HAPLN1 levels had a positive correlation with the inflammatory cytokines such as IL-6 (**Figure 6H**) and the modulators of the ECM structure, including TGF-β and fibronectin (**Figures 6I, J**). Thus, HAPLN1 promotes the production of inflammatory cytokines, which plausibly could provide a molecular basis for its contribution to cell viability and mobility. In RA-FLSs, AMPK activation results in up-regulation of HAPLN1 levels and vice versa. Based on the effect of HAPLN1 on AMPK expression, along with the possible regulation of the HAPLN1 expression through the cAMP-PKA-(possibly, AMPK)-RUNX1/2 pathway in granulosa cells (32), we proposed that a negative feedback loop between HAPLN1 and AMPK expression exists.

### 2.4 Proteome and mRNA-Seq Analysis of HAPLN1 Functions in RA-FLSs

As the current molecular interaction network of HAPLN1 is barely barren, to get acquaintance with HAPLN1 functions in RA-FLSs from a more comprehensive view, proteomics and transcriptome analyses were done with rHAPLN1 treated or si-HAPLN1 transfected RA-FLSs to further investigate HAPLN1 functions in RA-FLSs.

#### 2.4.1 Proteomics Analysis

**Figure 7:**
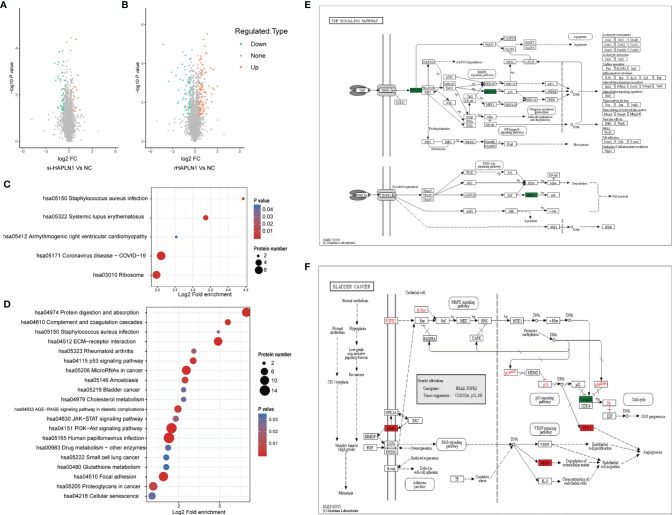
Proteomic analysis of si-HAPLN1- and rHAPLN1-treated RA-FLSs. **(A, B)** Among the quantifiable proteins, 14 were up-regulated and 47 were down-regulated by si-HAPLN1 transfection. There were 101 proteins up-regulated, and 82 were down-regulated by rHAPLN1 treatment compared to NC. **(C)** KEGG enrichment analysis shows the pathways associated with DEPs of si-HAPLN1-treated RA-FLSs. **(D)** KEGG enrichment analysis shows the top 20 pathways associated with DEPs of rHAPLN1-treated RA-FLSs. **(E)** DEPs of si-HAPLN1-treated RA-FLSs enriched in the TNF signaling pathway. **(F)**. DEPs of rHAPLN1-treated RA-FLSs enriched in the bladder cancer pathway. Targets in red blocks represent up-regulation, in green blocks represent down-regulation.

We identified 443,973 matched spectra and 4184 quantifiable proteins in RA-FLSs from NC, si-HAPLN1, and rHAPLN1 groups (**Supplementary Table S5**). Principal component analysis (PCA) indicated a high level of aggregation between duplicated samples, demonstrating the quantitative reproducibility of experiments (**Supplementary Figure S6**). Among the identified proteins, 14 were up-regulated and 47 were down-regulated after si-HAPLN1 transfection. Besides, 101 proteins were up-regulated and 82 were down-regulated after rHAPLN1 treatment as compared to NC (**Figures 7A, B**). Compared to the control siRNA-treated RA-FLSs (NC group) and as shown by KEGG enrichment analysis, differentially enriched proteins (DEPs) in si-HAPLN1-treated RA-FLSs were enriched in pathways including *Staphylococcus aureus* infection, systemic lupus erythematous (SLE), cardiomyopathy, COVID-19, and ribosome (**Figure 7C**). RA-FLSs treated with rHAPLN1 were enriched in pathways including protein digestion, *S. aureus* infection, ECM-receptor interaction, RA, p53 signaling pathway, cholesterol metabolism, PI3K-Akt signaling pathway, JAK-STAT signaling pathway, and pathways for various cancers (**Figure 7D**). More specifically, through clustering analysis, the down-regulated DEPs of the si-HAPLN1 group were enriched in *S. aureus* infection, SLE, TNF signaling pathway, COVID-19, and ribosome. Therefore, si-HAPLN1 treatment mostly results in DEPs that participate in down-regulating proteins related to inflammation. On the other hand, in the rHAPLN1-treated RA-FLSs, the down-regulated DEPs involved are in cellular senescence, drug metabolism, p53 signaling pathway, glutathione metabolism, RA, and cytokine-cytokine receptor interactions; the up-regulated DEPs involved are in cytochrome P450, malaria, platelet activation, JAK-STAT signaling pathway, PI3K-Akt signaling pathway, PPAR signaling pathway, human papillomavirus infection, relaxin signaling pathway, chemical carcinogenesis, microRNAs in cancer, cholesterol metabolism, amoebiasis, focal adhesion, and ECM-receptor interaction (**Supplementary Figure S7**). Based on these results, it is clear that rHAPLN1 treatment mainly leads to pro-inflammation, activation of metabolism, and carcinogenesis. However, pathway enrichment analysis also indicated cell adhesion reinforcement that confirms our transwell and wound healing assays.

As an example, we took the TNF signaling pathway affected by si-HAPLN1 transfection for description of our proteomics results. Tumor necrosis factor receptor type 1-associated death domain protein (TRADD) and mitogen activated protein kinases (MKK3/6), known for their role in apoptosis and cell survival were found to be down-regulated (**Figure 7E**). In rHAPLN1-treated RA-FLSs, up-regulation of MMPs and down-regulation of CyclinD1 were observed (**Figure 7F**). This is in accordance with our results obtained with HAPLN1^OE^ transfection in RA-FLSs (**Figures 5B** **, 6C, D**). An up-regulated multifunctional matrix glycoprotein thrombospondin-1 (TSP-1) together with matrix metalloproteinases (MMPs) suggest an increased level of angiogenesis, which is a classical pathology feature of pannus in RA (33). Up-regulated expression of collagen within the ECM-receptor interaction pathway (**Supplementary Figure S7**) is in accordance with the reduced expression of collagen II gene after transfection with si-HAPLN1 and with up-regulated expression of ACAN after transfection with HAPLN1^OE^ (**Figures 5C, D**). Proteomics results indicate upregulation of the expression of collagen, laminin, and thrombospondin (**Supplementary Figure S8**), which are associated with cell migration (34, 35).

Notably, rHAPLN1 up-regulated DEPs within various metabolism pathways. High demands for energy and biosynthetic precursors are well known in the pathogenic nature of RA (36). CYP1B1, glutathione S-alkyltransferase (EC: 2.5.1.18), cytochrome P450 (EC: 1.14.14.1), and aldehyde dehydrogenase (EC: 1.2.1.5) were up-regulated in the metabolism of xenobiotics *via* the cytochrome P450 pathway (**Supplementary Figure S9**). These four enzymes were reported to participate in the development of various cancers (37–40). Although these targets have not been well investigated in RA, detection of a higher metabolic level, along with the up-regulated expression of pro-inflammatory DEPs, suggest the effects of rHAPLN1 on the viability of RA-FLSs.

#### 2.4.2 mRNA Sequencing Analysis

**Figure 8:**
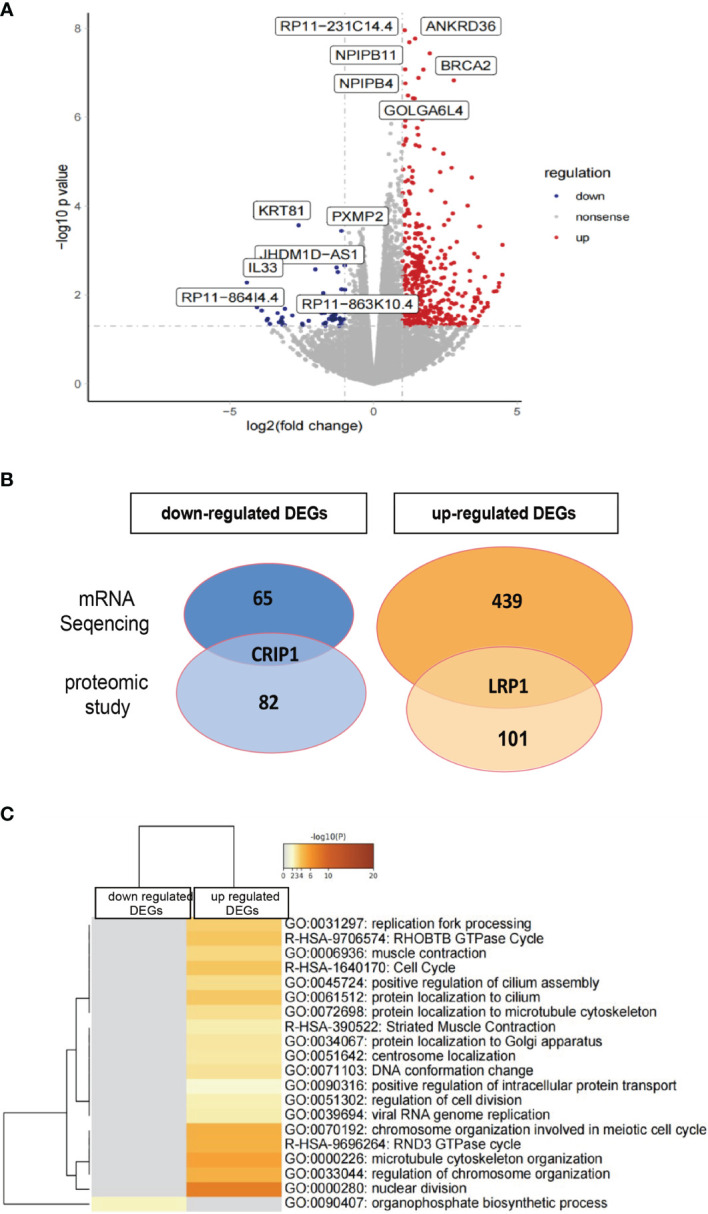
Transcriptome analysis of rHAPLN1-treated RA-FLSs. **(A)** Among the 504 DEGs detected, 439 were up-regulated and 65 were down-regulated. **(B)** Two DEGs detected (up-regulated LRP1 and down-regulated CRIP1) were in accordance with the proteomic studies. **(C)** Metascape analysis of DEGs demonstrated an enrichment of various pathways including replication fork processing, GTPase cycle, cell cycle, and cell division.

mRNA sequencing analysis was done with rHAPLN1- or PBS-treated RA-FLSs. Principal component analysis (PCA) showed a high level of aggregation between duplicated samples, suggesting the quantitative reproducibility of experiments (**Supplementary Figure S10**). Among the 504 differentially expressed genes (DEGs), 439 were up-regulated with the top 6 genes being RP11-231C14.4 (an uncharacterized gene), ANKRD36, NPIPB11, NPIPB4, BRCA2, and GOLGA6L4. At the same time, 65 genes such as KRT81, PXMP2, JHDM1D-AS1, and IL33 were down-regulated (**Figures 8A, B**). Two DEGs (up-regulated LRP1 and down-regulated CRIP1) were in accordance with the results found in our proteome analysis (**Figure 8B**). Metascape pathway analysis of 439 up-regulated DEGs treated by rHAPLN1 showed main enrichment of genes in the GTPase cycle, cell cycle, and regulation of cell division (**Figure 8C**). KEGG analysis showed enrichment in herpes simplex virus 1 infection, hypertrophic cardiomyopathy, and others (**Supplementary Figure S11**). Moreover, GSEA analysis was performed to compare rHAPLN1 and PBS groups. The rHAPLN1 group was found to positively associate with the pathways of extracellular matrix structural constituents, alpha actin binding, metalloaminopeptidase activity, proteoglycan binding, focal adhesion, regulation of protein exit from endoplasmic reticulum, lipid translocation, regulation of androgen receptor signaling pathway, retrograde axonal transport, dendritic spine development, peptide cross linking, and insulin-like growth factor receptor signaling pathway (**Supplementary Figure S12**). Although only 2 overlapping targets were identified in our proteomics study, these enriched functional pathways were in accordance with proteomics analysis and could potentially explain the effects of HAPLN1 on the proliferation, migration, and apoptosis of RA-FLSs.

## 3 Discussion

HAPLN1, discovered 50 years ago, has a wide range of physiological effects with an important contribution to cartilage formation and homeostasis as well as to the regulation of the development of the central nervous system (41). Besides HAPLN1, the HAPLN family includes paralogs of HAPLN2, HAPLN3, and HAPLN4, all of which related pathways are Phospholipase-C Pathway and Integrin Pathway. Annotations related to these genes include extracellular matrix structural constituent and hyaluronic acid binding. They constitute a HAPLN family (42, 43). HAPLN1 interacts with the globular domains of hyaluronic acid and proteoglycans, such as aggrecan, versican, and α-trypsin inhibitor in the ECM, to form a stable ternary complex and contributes to the compression resistance and shock absorption of the joints (44). During the process of chondrogenesis and differentiation of human mesenchymal stem cells (hMSCs), HAPLN1 expression reached its peak level between 6 and 12 days (45). Perinatal mice with inactivated HAPLN1 developed lethal achondroplasia, with their extremities and vertebral cartilage lacking proteoglycan deposition and having a reduced number of hypertrophic chondrocytes (46). In addition, HAPLN1 was shown to have the properties of an oncogene contributing to an increased susceptibility to lung cancer (47), aggressiveness of hepatocellular carcinomas (48), and drug resistance to multiple myeloma (49).

We have reported HAPLN1 as one of the most obviously up-regulated DEGs in RA-FLSs and upon activation of AMPK by metformin HAPLN1 secretion has increased in RA-FLSs (9). Based on AMPK functions and the use of metformin in RA, we hypothesized that an increase in the levels of HAPLN1 in RA-FLSs could help protect the joints (9). In this study, we demonstrated an increase in HAPLN1 expression in the synovium and plasma samples from RA patients. Over-expression of HAPLN1 with a plasmid vector or treatment with rHAPLN1 increased the proliferation but decreased apoptosis of RA-FLSs. Although si-HAPLN1 transfection did not show any effect on proliferation, it induced apoptosis of RA-FLSs significantly. So, HAPLN1 seems to be an oncogenic gene, and it could activate the viability of RA-FLSs.

Apart from hyaluronic acid and proteoglycans, interactions between HAPLN1 and other molecules have been investigated. For example, TNF-ɑ-activated mitogen-activated kinase (MEK) in chondrocytes regulates the expression of HAPLN1, and controls the catabolism and anabolism of the extracellular matrix of chondrocytes (50). In multiple myeloma cells, HAPLN1 can activate the NF-ƙB pathway to acquire resistance to bortezomib (49). In granulosa cells, HAPLN1 can potentially promote the PKA-RUNX1/RUNX2 pathway (9, 32). In this study, upon silencing HAPLN1, pro-inflammatory factors such as TNF-ɑ, MMPs, and IL-6 as well as structure-related molecules such as TGF-β, fibronectin, and ACAN were down-regulated. Conversely, HAPLN1^OE^-treated RA-FLSs showed up-regulation of TNF-ɑ, MMPs, IL-6, and ACAN expression. In untreated RA-FLSs, the relative mRNA expression of HAPLN1 was positively associated with that of TGF-β and IL-6. The Ki-67 has been widely used as a proliferation marker for most human tumor cells (51). Its expression was decreased after si-HAPLN1 transfection but increased after HAPLN1^OE^ treatment. Based on these findings and the current knowledge, HAPLN1 is expected to be able to promote pro-inflammatory secretory phenotypes and to contribute to the regulation of structural molecules in cells.

AMPK and its related pathway have been broadly investigated with participation in glucose metabolism and inflammation reaction, generally in an inhibitory way (31), specifically, inflammatory cytokines such as TNF-ɑ, IL-6, IL-17, NF-ƙB, and MMPs are directly or indirectly inhibited (52, 53). Relative mRNA expression of AMPK-ɑ and HAPLN1 in untreated RA-FLSs showed a positive correlation, which is consistent with the observation made on plasma samples from RA patients. Thus, in this study, we intended to examine whether AMPK is affected by HAPLN1 expression to clarify its effect on inflammation. However, both si-HAPLN1- and HAPLN1^OE^-treated RA-FLSs down-regulated AMPK-ɑ expression at the mRNA level, but not the protein level, of pAMPK-ɑ. This suggests the presence of a complex feedback circle between AMPK-ɑ and HAPLN1. AMPK and its phosphorylation levels are recognized to be especially vulnerable to variations in the metabolism status such as the AMP/ATP ratio (54). Based on our omics study, we proposed that HAPLN1 turns cells into a more hyperactive and hypermetabolism status. So, after silencing HAPLN1, a lower metabolic status of higher AMP/ATP ratio results in an increased level of pAMPK-ɑ. However, a higher metabolic status accompanied by inflammation lowered the AMP/ATP ratio as represented by up-regulated pAMPK-ɑ. Such a hypothesis needs further experimental validation.

Furthermore, Cyclin D1 is involved in the regulation of cell proliferation during the G1 phase of the cell cycle. Given the frequent over-expression of Cyclin D1 in cancer cells, its expression appears to be closely linked with carcinogenesis (55). Cyclin D1 has a central role in mediating invasion and metastasis of cancer cells by controlling Rho/ROCK signaling and matrix deposition of thrombospondin-1 (56). We designed to check this target to verify possible promotion of cell viability by HAPLN1. In this study, however, the mRNA expression levels of Cyclin D1 in RA-FLSs was significantly decreased by HAPLN1^OE^ treatment, which might possibly explain its inhibitory ability on RA-FLS migration. There is a dilemma in clarifying the role of HAPLN1 in RA-FLSs viability by functional studies in the cancer research field because an increased level of HAPLN1 seems to be associated with a higher degree of aggressiveness, leading to stemness of various cancers (47, 48, 57) while achieving robust ECM restrictions on metastasis of cancer cells (19, 20).

Proteomic and mRNA-seq results showed the function of HAPLN1 in RA-FLSs from a holistic view. With highly significant changes observed in the expression of DEPs and DEGs, it is plausible to consider the involvement of HAPLN in a complex network of signaling pathways. Proteomic analysis suggested si-HAPLN1-transfected RA-FLSs were enriched in pro-inflammatory pathways with down-regulated DEPs. It is not strange that the mRNA level and the protein level seem to have a low correlation, as the multi-step process of gene expression involves transcription, translocation, and turnover of mRNAs and proteins (58). Although only 2 targets overlapped with proteomic and transcriptional studies with rHAPLN1-treated RA-FLSs, the omics study reflected activation of inflammation, proliferation, an increase in cell adhesion, and strengthening of ECM functions. These findings were confirmed by the molecular network consisting of MMPs, IL-6, Ki-67, TGF-β, and cyclin D1 as shown by qPCR and Western blot analysis. Genes such as ANKRD36 (59), BRCA2 (60), and GOLGA6L4 (61), which were most up-regulated upon rHAPLN1 treatment, were all reported as oncogenic targets. In consistence with the reported close relation between HAPLN1 and AMPK levels, HAPLN1 seems to be involved in the metabolism of RA-FLSs. Spontaneously resolving joint inflammation in RA was reported to be dependent on the metabolic agility of FLS (62), and increased levels of SUMOylation links metabolic and aggressive phenotype of RA-FLSs (63). Therefore, altering metabolic changes might be a key to developing joint-protective strategies in RA-FLSs (64) and more research is required to decipher the complex network of HAPLN1 functions contributing to the altered metabolic status of RA-FLSs.

In conclusion, HAPLN1 accelerates proliferation and reduces apoptosis of RA-FLSs to form a pathological pannus, mimicking the aggressive feature of cancer cells. Based on physiological development and oncology studies, HAPLN1 seems to be an oncogene but having an opposing feature on cell adhesion and inhibition of migration. By combining biological experiments and the observations made from proteomics and mRNA sequencing analysis, our results suggest HAPLN1 as a pathogenic factor in RA. Future in-depth studies, especially those related to animal experiments, are mandatory for better understanding of the role of HAPLN1 in RA.

## 4 Material and Methods

### 4.1 Patients’ Characteristics and Samples

We used blood samples from 61 RA and 20 OA patients and 12 age- and gender-matched healthy controls (HC) for measuring HAPLN1 levels by ELISA. The mean disease course of RA patients was 6.39 years (ranging from 0.2 to 30.0 years). Synovium samples were collected by arthroscopic surgery done with 20 RA and 17 OA patients and used for immunohistochemical (IHC) staining of HAPLN1. The inclusion and exclusion criteria and general information such as age, gender, disease activity, and disease course reported earlier (9) are summarized in **Supplementary Tables S1** **, 2**.

### 4.2 Enzyme Linked Immunosorbent Assay (ELISA)

Blood samples of HC, OA, and RA patients were centrifuged after standing at room temperature for 2 h, at 1500 g for 10 min to collect the plasma. The HAPLN1 levels were detected by ELISA (RayBiotech, US). Plasma AMPK levels in RA patients were also evaluated by ELISA according to the manufacturer’s protocol (Jianglaibio, China). The SuPerMax 3000FA absorbance microplate reader (Flash Co. Ltd., China) was used to read the optical density (OD) values at 450 nm and concentrations of specific proteins were calculated based on the standard curve.

### 4.3 Immunohistochemical (IHC) Staining for HAPLN1

Synovium samples collected from 20 RA and 17 OA patients for IHC staining of HAPLN1 were prepared as reported earlier (9). Rabbit monoclonal anti-HAPLN1 antibody (Abcam, US) was added as the primary antibody (1:50) and incubated for 2 h at 37°C. Biotin-conjugated goat anti-rabbit antibodies (ZSGB-Bio, China), streptavidin-peroxidase conjugate, and diaminobenzidine were used as the detecting system. IHC-stained sections were semi-quantified under a microscope. The staining intensity was counted as none (0 points), weak positive (1+), moderate positive (2+), and strong positive (3+). The percentage of positive cells was obtained to calculate the H-Score. The range of H score for each slice was between 0 and 300. The formula of the H-Score is as follows (65):


H sore=(% at weak positve) ×1+(% at morderate positve)×2+(% at strong positve) ×3

### 4.4 Isolation and Culture of RA-FLSs

Primary RA-FLSs were acquired from 3 untreated RA patients. Isolation and culture of RA-FLSs were reported as before (9). Briefly, FLSs were isolated by enzyme digestion and subsequently cultured in Dulbecco’s modified essential medium (DMEM) containing 10% fetal bovine serum (FBS, Invitrogen) and antibiotics (penicillin and streptomycin) at 37°C with 5% CO_2_. Cells cultured between passages 4 and 9 were used in this study.

### 4.5 Small Interfering RNA (siRNA) HAPLN1 Preparation and Transfection

RA-FLSs at 60-70% confluency were transfected with siRNAs (Ribobio Company, China) at 50 nM with Lipofectamine™ 3000 reagent (Invitrogen, US). The following siRNA sequences were used: control siRNA (confidential sequence information) and 3 siRNAs of HAPLN1, si-1 (5’-AGGGTAGAGTGTTTCTGAA-3’), si-2 (5’-CCTGGAAAATTCTCGGATA), and si-3 (5’-ACCTCACTCTGGAAGATTA-3’). The three siRNAs effectively silenced HAPLN1 expression (**Supplementary Figure S2A**), and si-1 was selected randomly and used in subsequent studies. The negative control (NC) group denoted control siRNA transfection of RA-FLSs in the experiment.

### 4.6HAPLN1 Over-Expression Vector Preparation and Transfection

For HAPLN1^OE^ RA-FLSs experiments, HAPLN1 over-expression plasmid and its control were constructed and packaged by Ubigene Biosciences (Guangzhou, China). The stbl3 strain plasmid cytomegalovirus vector-infected cells were cultured in LB medium (QDRS Biotec, China) with 100 g/ml of ampicillin under 37°C, 225 rpm for 24 h. The HAPLN1^OE^ plasmid vector and its negative control (NC) were then isolated with the Genopure Plasmid Maxi Kit (Roche, US). RA-FLS at 60-70% confluency was transfected with HAPLN1^OE^ vector or its negative control with Lipofectamine™ 3000 reagent (Invitrogen). The effects of HAPLN1^OE^ plasmid vector are shown in **Supplementary Figure S2B**.

### 4.7 MTT Assay

MTT assay was used to ascertain FLSs viability transfected with si-HAPLN1, HAPLN1^OE^, or their corresponding NC, or treated with rHAPLN1 (recombinant human HAPLN1 protein, Abcam, US) at different concentrations (0, 25, and 50 ng/ml). FLSs samples (si-HAPLN1 vs. its negative control, HAPLN1^OE^ vs. its negative control, or treated with different concentrations of rHAPLN1) digested using 0.25% pancreatin were transferred to 96-well plates with 3-5 × 10^3^ cells/well. At different time points (24, 48, and 72 h), the viability of the cells was measured using the MTT assay kit (Abcam).

### 4.8 CCK-8 Assay

Cell viability after transfection with si-HAPLN1, HAPLN1^OE^, or their respective controls, or treated with different concentrations of rHAPLN1, was determined using the Cell Counting Kit-8 (CCK-8, Molecular Technology, Japan) assay.

### 4.9 TUNEL Assay

RA-FLSs transfected with si-HAPLN1, HAPLN1^OE^, or their corresponding controls, or treated with rHAPLN1 (0 or 50 ng/ml), were digested and transferred to 6-well plates with 2-3 × 10^5^ cells/well, cultured for 48 h, and stained by the One Step TUNEL Apoptosis Assay Kit (Beyotime, China). The apoptosis rate was calculated under a fluorescence microscope (Leica, Germany) with the excitation wavelength at 550 nm (Cy3) and the emission wavelength at 570 nm (red fluorescence).

### 4.10 Flow Cytometry for FLSs Apoptosis

FLSs apoptosis treated with rHAPLN1 was measured using the Annexin V-FITC/PI Cell Apoptosis Detection Kit (Vazyme, China) by flow cytometry. After treatment of FLSs with rHAPLN1 (0 or 50 ng/ml) for 48 h in 6-well plates, the cells were collected (3×10^5^/well), washed twice with PBS, re-suspended in 500 µl 1 × binding buffer, mixed with Annexin-V-fluorescein isothiocyanate (FITC, 5 µl) and propidium iodide (PI, 5 µl), and analyzed using a flow cytometer (BD FACSCantoTM II, US). The scatter diagram was distributed as follows: Q3, healthy cells (FITC-/PI-); Q2, apoptotic cells at an advanced stage (FITC+/PI+); and Q4, apoptotic cells at an early stage (FITC+/PI-). The apoptosis rate was calculated as a ratio of apoptotic cells in P2 (Q4 + Q2).

### 4.11 Wound Healing Assay

Wound healing assay was conducted to evaluate the migration capacity of FLSs transfected either with si-HAPLN1, HAPLN1^OE^, or their corresponding controls, or treated with different concentrations (0, 25, and 50 ng/ml) of rHAPLN1. FLS samples (si-HAPLN1 vs. its negative control, HAPLN1^OE^ vs. its negative control, or treated with different concentrations of rHAPLN1) were transferred to 6-well plates with 3 × 10^5^ cells/well and cultured with serum free-RPMI 1640 medium. At different time points, the migrating ability of the cells was measured by using the wound healing assay as previously reported (9).

### 4.12 Transwell Assay

Transwell assay was performed to evaluate the migration capacity of FLSs transfected with si-HAPLN1, HAPLN1^OE^, or their respective controls, or treated with rHAPLN1. FLSs in each set of experiments were re-suspended after culturing for 24 h. Transwell assay was conducted as previously described (9).

### 4.13 Quantitative Real-Time Polymerase Chain Reaction (qPCR)

Total RNA from FLSs transfected with si-HAPLN1, HAPLN1^OE^, or their respective controls, or treated with rHAPLN1, was prepared using TRIzol^®^ Reagent (Thermo Scientific, US) and quantified using Qubit (Thermofisher, US). RNA was reverse transcribed into cDNA using PrimeScript™ RT Master Mix (Takara, Japan). The reaction mixture contained 5 ml of 2 × TB Green Premix Ex Taq II (Takara, Japan), 3 ml of nuclease-free water, 1 ml of cDNA, 0.4 ml of each gene-specific primer, and 0.2 l of ROX reference dye. The qRT-PCR analysis was performed using the Applied Biosystems ViiA™ 7 Real-Time PCR System (Thermofisher, US). Each value represented an average from three independent biological replicates. GAPDH gene expression was used for data standardization. The fold change was calculated using the 2-ΔΔCt method. Primers of GAPDH, AMPK-ɑ, TNF-ɑ, IL-6, TGF-β, ACAN, fibronectin, collagen II, MMP1, MMP3, MMP9, Cyclin-D1, and Ki-67 are given in **Supplementary Table S3**.

### 4.14 Automated Western Blot Analysis

Total proteins from FLSs transfected with si-HAPLN1, HAPLN1^OE^, or their respective controls for 48 h were extracted with Cell Lysis Buffer (Cell Signaling, US). Their concentration was measured using the BCA Protein Assay Kit (Merck, US). Relative changes in HAPLN1, pAMPK-α, IL-6, TNF-α, MMP1, MMP3, and MMP9 protein levels were determined. Expression of β-tubulin was selected as an internal reference. Capillary electrophoresis and Western blot analysis were carried out using reagents provided in the kit in accordance to the instructions provided by the user manual (ProteinSimple WES, US) as previously reported (9). Rabbit anti-HAPLN1 antibody (Abcam, US), rabbit anti-TNF-α, AMPK-α, pAMPK-α, MMP-1, MMP-3, IL-6, Cyclin D1, and β-tubulin specific mAbs (Cell Signaling, US) were used (1:100). Goat anti-rabbit secondary antibodies were provided by the ProteinSimple WES kit and applied as instructed. Data were analyzed using an in-built Compass software SW 4.0. The truncated and full-length target protein intensities (area under the curve) were normalized to that of the tubulin peak. In most of the figures, electropherograms were represented as pseudo-blots, generated using Compass software.

### 4.15 Statistical Analysis

Statistical analysis was performed using GraphPad Prism 8.0 software. All the data were given as mean ± SD. Differences between two groups were evaluated for statistical significance using Student’s t-test. One-way ANOVA with Tukey’s multiple comparisons test was used to evaluate the differences among three or more groups. Correlations were evaluated using Liner regression and correlation test. *p* < 0.05 was considered statistically significant.

### 4.16 Proteomics Analysis

Label-free proteomics study was applied to FLSs transfected with si-HAPLN1 or treated with rHAPLN1 (50 ng/ml) and their controls for 48 h (management of each group is given in **Supplementary Table S4**) by PTMBiolabs, Inc. (Hangzhou, China). Each concentration was tested with 3 biological replicates. Cell samples were processed as reported earlier (66). LC−MS/MS proteomics analysis was performed on an EASY-nLC 1000 ultra-performance liquid chromatography (UPLC) system, followed by MS/MS using Q Exactive Plus (ThermoFisher Scientific, US) coupled online to the UPLC system. The MS/MS data were retrieved by the Maxquant search engine (v1.6.6.0). A human database was searched (Swiss-Prot). The decoy database anti-library was used to reduce the false positive rate (FDR). The FDR was adjusted to < 1%, and the minimum score for modified peptides was set > 40. Proteins with a fold-change ≥1.50 or ≤0.67 between si-HAPLN1, rHAPLN1, and their controls were considered as expression significant. Based on the protein sequence alignment method, the protein domain functions were defined by InterProScan (http://www.ebi.ac.uk/interpro/). Functional annotation enrichment of DEPs was performed by KEGG analysis. The enrichment significance was identified as p < 0.05 in the Fisher’s exact test and q < 0.05 in the Benjamini-Hochberg procedure.

### 4.17 High-Throughput mRNA Sequencing Analysis

High-throughput RNA sequencing was performed using FLSs after treatment with rHAPLN1 (0 and 50 ng/ml) for 48 h. Each concentration was tested three times. RNA-seq analysis was performed by Seqhealth Technology Co., Ltd (Wuhan, China). Total RNA (2 g) was used for stranded RNA sequencing library preparation using KCTM Stranded mRNA Library Prep Kit for Illumina^®^ (Seqhealth Co., Ltd. China). PCR products corresponding to 200-500 bps were enriched, quantified, and sequenced with Novaseq 6000 sequencer (Illumina), PE150 model. Raw sequencing data were first filtered by Trimmomatic (v. 0.36). Low-quality reads were discarded. The reads contaminated with adaptor sequences were trimmed. Clean data were mapped to the human reference genome from UCSC (https://genome.ucsc.edu/) using STRA software (v. 2.5.3a) with default parameters. Reads mapped to the exon regions of each gene were counted by feature Counts (Subread-1.5.1; Bioconductor) and then RPKMs were calculated. DEGs between groups were identified using the edgeR package (v. 3.12.1) in R studio software (version 3.6). A p-value cut-off of 0.05 and fold-change cut-off of 2.0 were used to judge the statistical significance of gene expression differences. The volcano plot was drawn with the ggplot2 package in R studio. Heatmaps of pathway enrichment analysis of DEGs were generated using Metascape (http://metascape.org) and a *P-*value less than 0.05 was statistically significant. KEGG enrichment analysis for DEGs was performed using KOBAS software (v. 2.1.1) with a p-value cut-off of 0.05. To compare transcriptome characteristics of rHAPLN1 with PBS groups, GSEA software (version 4.0.0) was used. Annotated pathway files (c5.go.bp.v7.4.symbols.gmt) were downloaded in the MSigDB database (http://www.gsea-msigdb.org/gsea/msigdb/collections.jsp). Pathways with a *P*-value less than 0.05 and false discovery rate (FDR) less than 0.2 were significantly enriched.

## Data Availability Statement

The RNA-seq data presented in this study have been deposited in the the NCBI Gene Expression Omnibus (GEO) database (https://www.ncbi.nlm.nih.gov/), under accession code GSE200597. The mass spectrometry proteomics data have been deposited to the ProteomeXchange Consortium (http://proteomecentral.proteomexchange.org) via the iProX partner repository with the dataset identifier PXD033131.

## Ethics Statement

The studies involving human participants were reviewed and approved by Ethics Committee of Shenzhen Peoples’ Hospital. The patients/participants provided their written informed consent to participate in this study.

## Author Contributions

Concept and design: YC and DZL. Experiment performance: YC, LYW, BJW, QYW. Acquisition, analysis and interpretation of data: YC, YJC and KSN. Drafting and revising the manuscript: YC, KSN, W-FL and BJW. All authors contributed to the article and approved the submitted version.

## Funding

The study was funded by the China Postdoctoral Science Foundation Project (No. 2021M701438); National Natural Science Foundation of China No. 81971464) and the National Key Research and Development Program (2019YFC0840600).

## Conflict of Interest

The authors declare that the research was conducted in the absence of any commercial or financial relationships that could be construed as a potential conflict of interest.

## Publisher’s Note

All claims expressed in this article are solely those of the authors and do not necessarily represent those of their affiliated organizations, or those of the publisher, the editors and the reviewers. Any product that may be evaluated in this article, or claim that may be made by its manufacturer, is not guaranteed or endorsed by the publisher.
