# Supplementary material for: HAPLN1 Affects Cell Viability and Promotes the Pro-Inflammatory Phenotype of Fibroblast-Like Synoviocytes
Source: Front Immunol. 2022 Jun 2;13:888612. doi: 10.3389/fimmu.2022.888612 (PMC9202519; doi:10.3389/fimmu.2022.888612)
Supplement: Supplementary file 1 [file DataSheet_1.docx]

Supplementary Material

**Supplementary Table 1. Patients’ details, who donated synovium for IHC staining studies (mean** ± **SD)**

| **General information** | **RA (n=20)** | **OA (n=17)** | ***P* value** |
| --- | --- | --- | --- |
| Male: female | 8:12 | 5:12 | > 0.05 |
| Age (years) | 50.5 ± 13.5 | 60.6 ± 8.9 | < 0.001 |
| ESR (mm/h) | 79.4 ± 46.9 | 39.4 ± 26.1 | < 0.001 |
| CRP (mg/L) | 27.3 ± 4.70 | 8.70 ± 4.30 | < 0.05 |

**Supplementary Table 2. General information of participants, who donated plasma (mean** ± **SD)**

| **General information** | **HC (n = 12)** | **OA (n = 20)** | **RA (n = 61)** | ***P* value** |
| --- | --- | --- | --- | --- |
| Gender (female: male) | 9:3 | 12:8 | 53: 17 | > 0.05 |
| Age (years) | 49.8 ± 15.6 | 53.5 ± 12.0 | 51.2 ± 12.6 | > 0.05 |
| ESR (mm/h) | NA | 37.8 ± 29.3 | 86.5 ± 50.5 | < 0.01 |
| CRP (mg/L) | NA | 5.13 ± 7.19 | 19.1 ± 13.63 | < 0.05 |

NA, not available.

**Supplementary Table 3. Primer sequences**

| **Gene name** | **5’ - 3’** | **3’ - 5’** |
| --- | --- | --- |
| GAPDH | CAATGACCCCTTCATTGACC | GACAAGCTTCCCGTTCTCAG |
| TNF-ɑ | GGCCCGACTATCTCGACTTTG | CGTTTGGGAAGGTTGGATGTT |
| IL-6 | CCTGAACCTTCCAAAGATGGC | TTCACCAGGCAAGTCTCCTCA |
| MMP1 | AAAATTACACGCCAGATTTGCC | GGTGTGACATTACTCCAGAGTTG |
| MMP3 | TGAAATTGGCCACTCCCTGG | GGAACCGAGTCAGGTCTGTG |
| MMP9 | TGTACCGCTATGGTTACACTCG | GGCAGGGACAGTTGCTTCT |
| AMPK-ɑ | TTGAAACCTGAAAATGTCCTGCT | GGTGAGCCACAACTTGTTCTT |
| ACAN | CCCCTGCTATTTCATCGACCC | GACACACGGCTCCACTTGAT |
| Cyclin-D1 | GCTGCGAAGTGGAAACCATC | CCTCCTTCTGCACACATTTGAA |
| Ki-67 | CGTCCCAGTGGAAGAGTTGT | CGACCCCGCTCCTTTTGATA |

**Supplementary Table 4. Management of NC, si-HAPLN1 and rHAPLN1**

| **Groups** | **si-RNA** | | **rHAPLN1** | |
| --- | --- | --- | --- | --- |
|  | si-Control | si-HAPLN1 | 0 ng/ml (PBS) | 50 ng/ml |
| NC | + | - | + | - |
| si-HAPLN1 | - | + | + | - |
| rHAPLN1 | + | - | - | + |

**Supplementary Table 5. Overview of protein identification**

| **Title** | **Number** |
| --- | --- |
| Total number of spectra | 1022232 |
| Number of matched spectra | 443973 |
| Peptides | 38576 |
| Unique peptides | 37344 |
| Identified proteins | 4944 |
| Quantifiable proteins | 4184 |

Note: 1. Total number of spectra includes number of total spectra and number of secondary spectra generated by mass detection; 2. Number of matched spectra includes the number of effective spectra and the number of spectra matching the theoretical secondary spectra; 3. Peptides include the number of identified peptides and the number of peptide sequences resolved by the matching result; 4. Unique peptides include the number of unique identified peptides and, the number of unique peptide sequences resolved by the matching result; 5. Identified proteins include the number of identified proteins; 6. Quantifiable proteins include quantitative protein numbers and the number of proteins quantified by specific peptides.


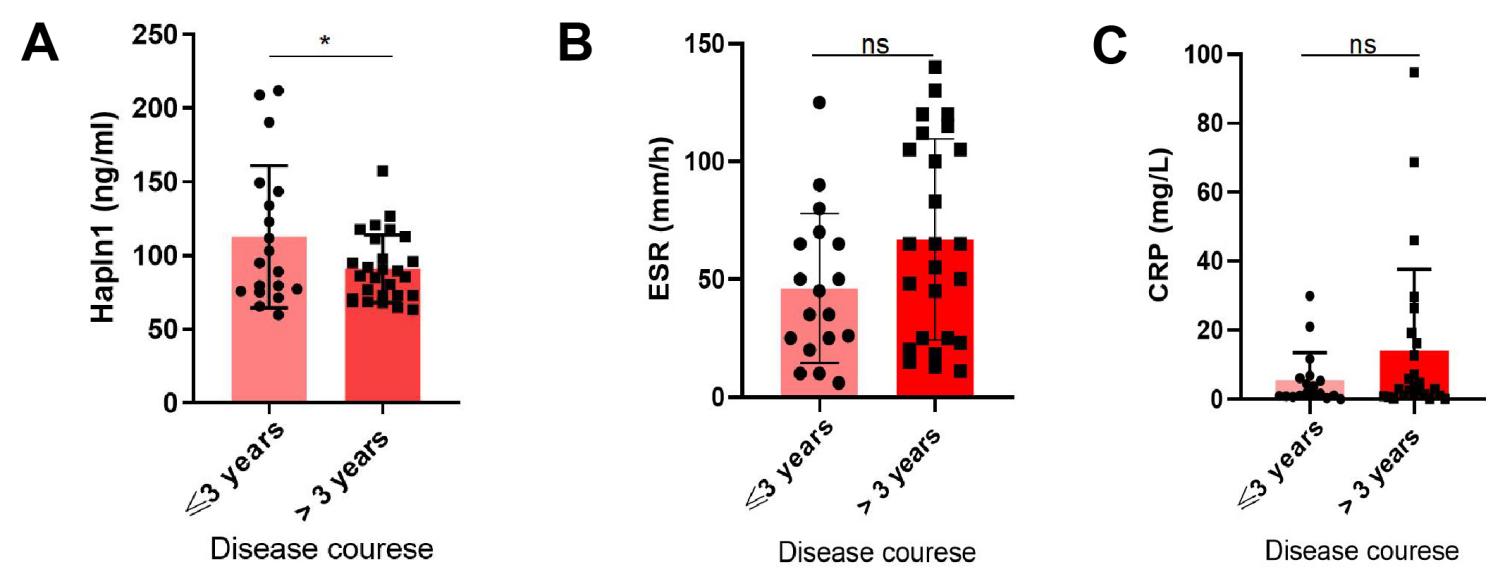


**Supplementary Figure 1.** (A) HAPLN1 in RA patients with less than 3 years of disease course history (n=20) were higher than those longer than 3 years (n=41). (B-C) No significant differences between RA patients with disease course less or longer than 3 years on ESR and CRP. *p < 0.05; ns, not significant.


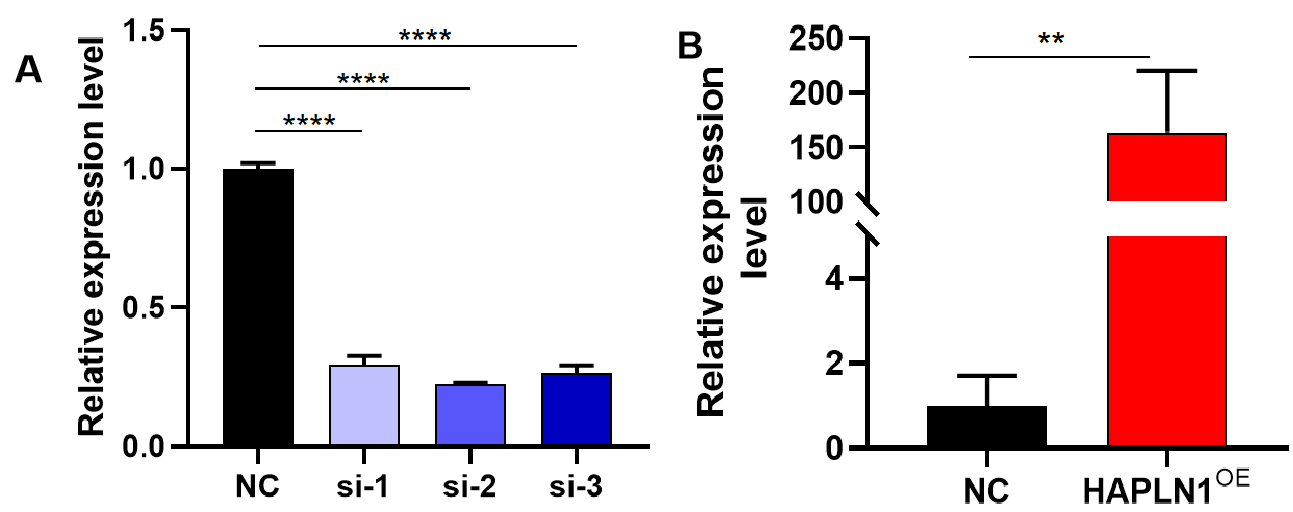


**Supplementary Figure 2.** Silencing or over-expression of HAPLN1 in RA-FLS. (A) All the three si-HAPLN1s designed and transfected showed significant inhibitory effect on the expression of HAPLN1 mRNA in RA-FLSs. (B) Over-expression plasmid vector (HAPLN1^OE^) has significantly up-regulated the expression of HAPLN1 mRNA in RA-FLSs. **p < 0.01; ****p < 0.00001. NC, negative control. NC in (A) is control siRNA of si-HAPLN1, NC in (B) is control plasmid vector of HAPLN1^OE^


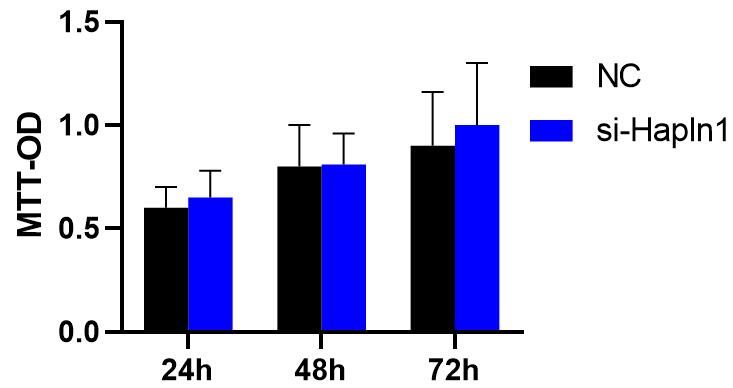


**Supplementary Figure 3.** Transfection of si-HAPLN1 in RA-FLSs did not show any significant effect on FLSs proliferation. NC, negative control is control siRNA of si-HAPLN1; si-HAPLN1, small interfering HAPLN1 RNA.


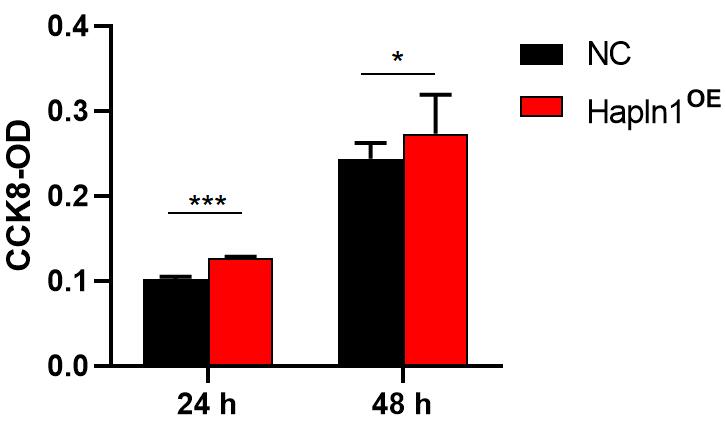


**Supplementary Figure 4.** HAPLN1 over-expression in RA-FLSs has significantly increased proliferation activity of FLSs. *p < 0.05; ***p < 0.001. NC, negative control is control plasmid vector of HAPLN1^OE^; HAPLN1^OE^, HAPLN1 over expression.


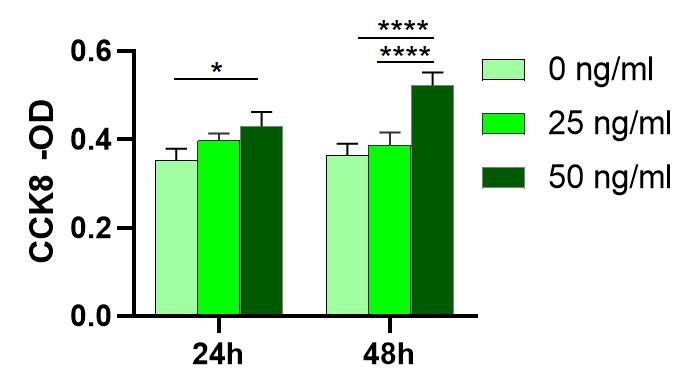


**Supplementary Figure 5.** FLSs proliferation was increased by rHAPLN1. *p < 0.05; ****p < 0.0001.


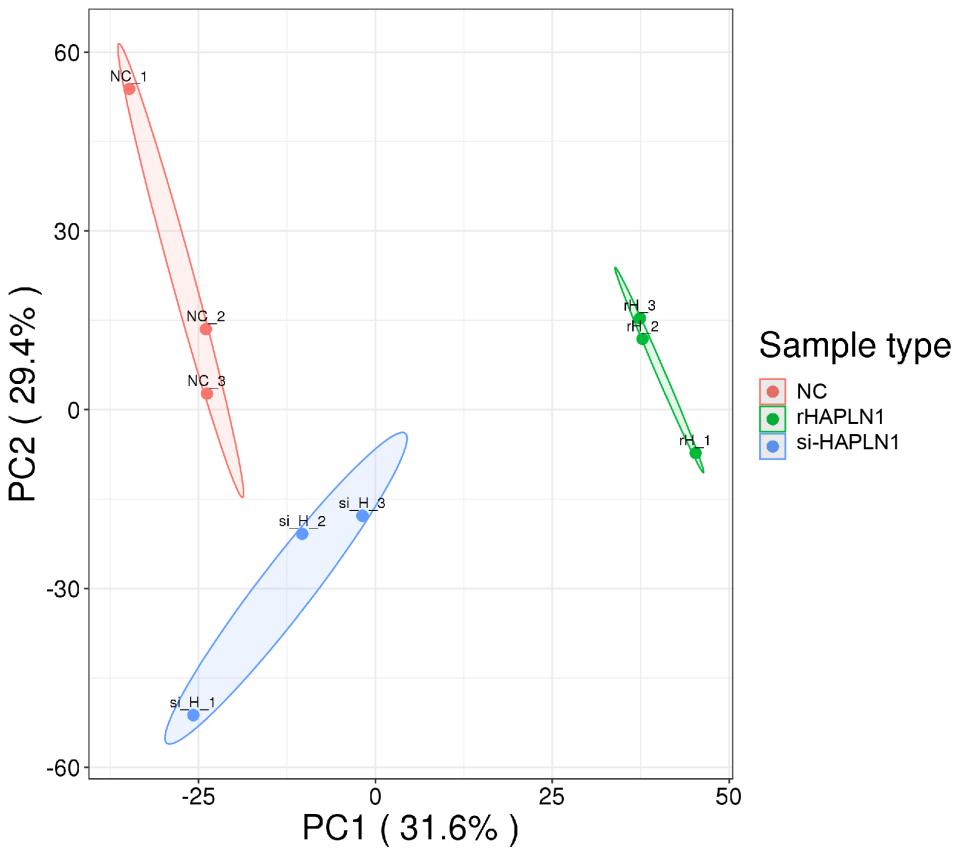


**Supplementary Figure 6.** Quantitative protein principal component analysis (PCA) of all the sample results are shown. Higher aggregation degree between the repeated samples indicates better quantitative repeatability. NC, negative control is control siRNA of si-HAPLN1.


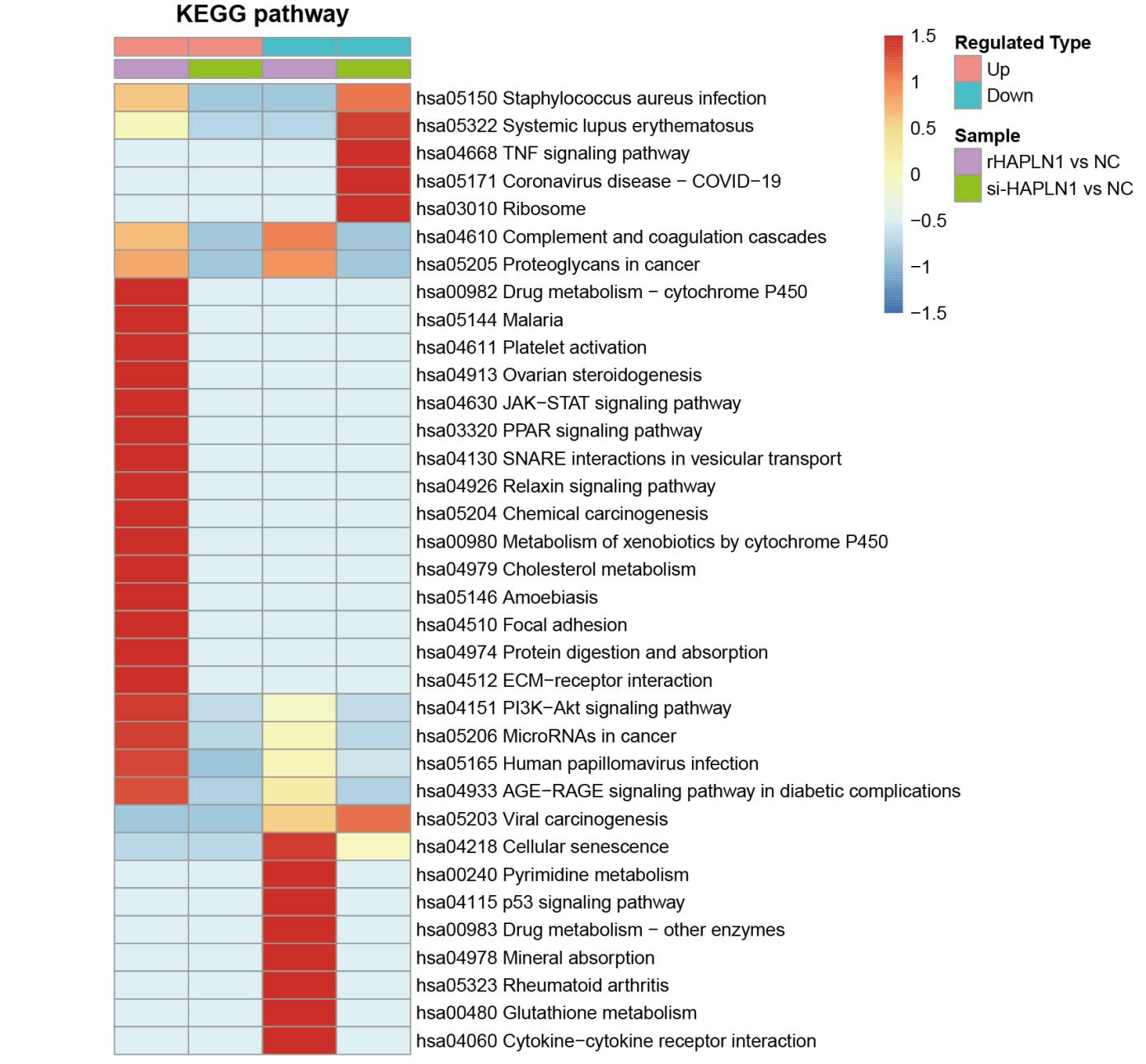
.

**Supplementary Figure 7.** Cluster analysis of the differentially expressed proteins (DEPs) in rHAPLN1 and si-HAPLN1 treated RA-FLSs. NC, negative control is control siRNA of si-HAPLN1.

After the DEPs in different comparison groups were subjected to GO classification, KEGG pathway and protein domain enrichment, we performed cluster analysis to find the functional correlation of the differentially expressed proteins in the comparison groups. Clustering method: The p-values obtained from the enrichment analysis (Fisher's exact test) used hierarchical clustering to cluster related features in different groups together and plotted as a heatmap. The horizontal direction of the heatmap represents the enrichment test results of different groups, and the vertical direction is the description of differentially expressed enrichment-related functions (GO, KEGG pathway, protein domain). The color blocks corresponding to the functional descriptions of the differentially expressed proteins in different groups indicate the degree of enrichment. Red represents strong enrichment and blue represents weak enrichment.


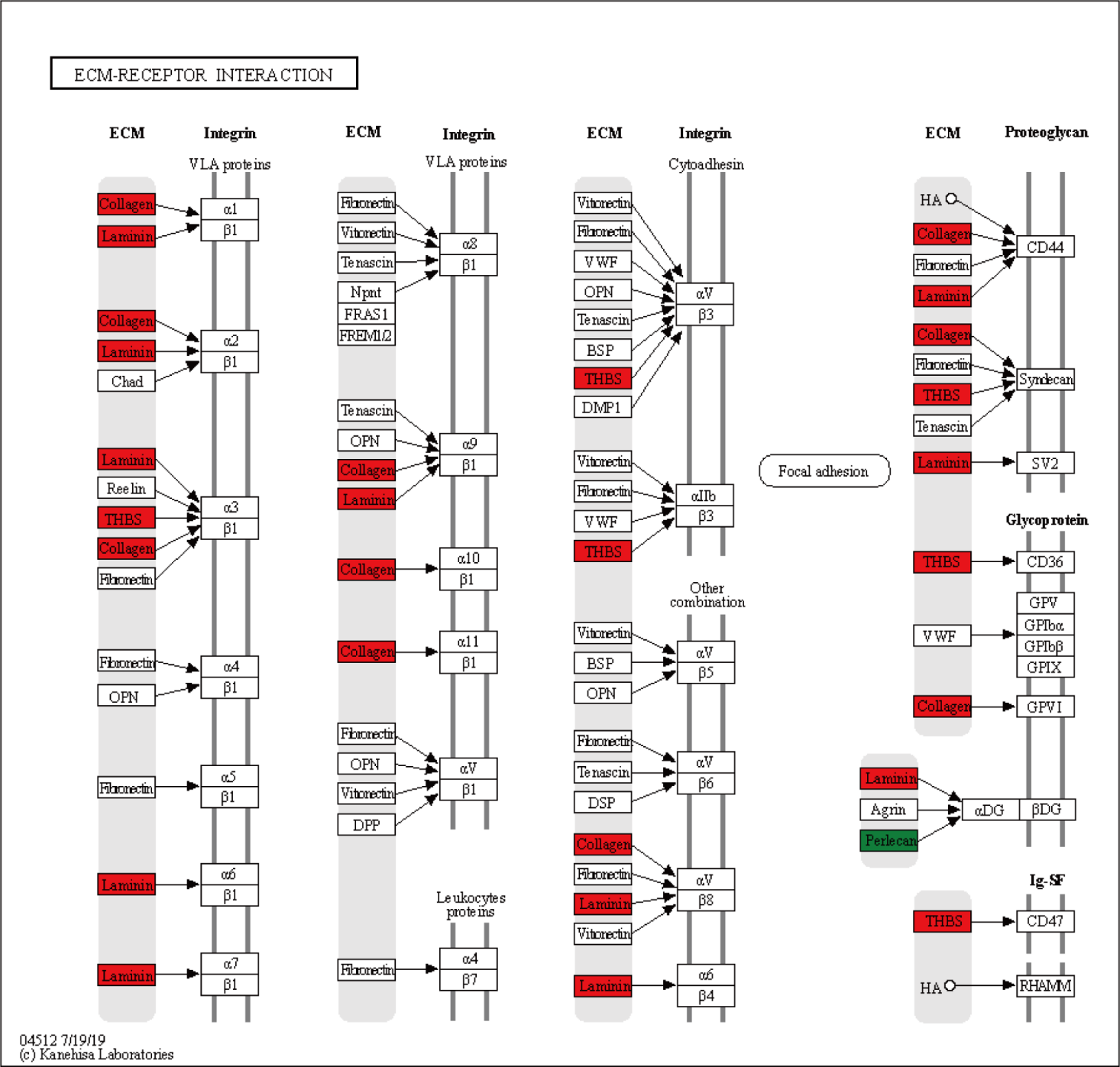


**Supplementary Figure 8.** DEPs that enriched in ECM-receptor interaction pathway of rHAPLN1 treated RA-FLSs. Targets in red blocks represent up-regulated, in green blocks represent down-regulated.


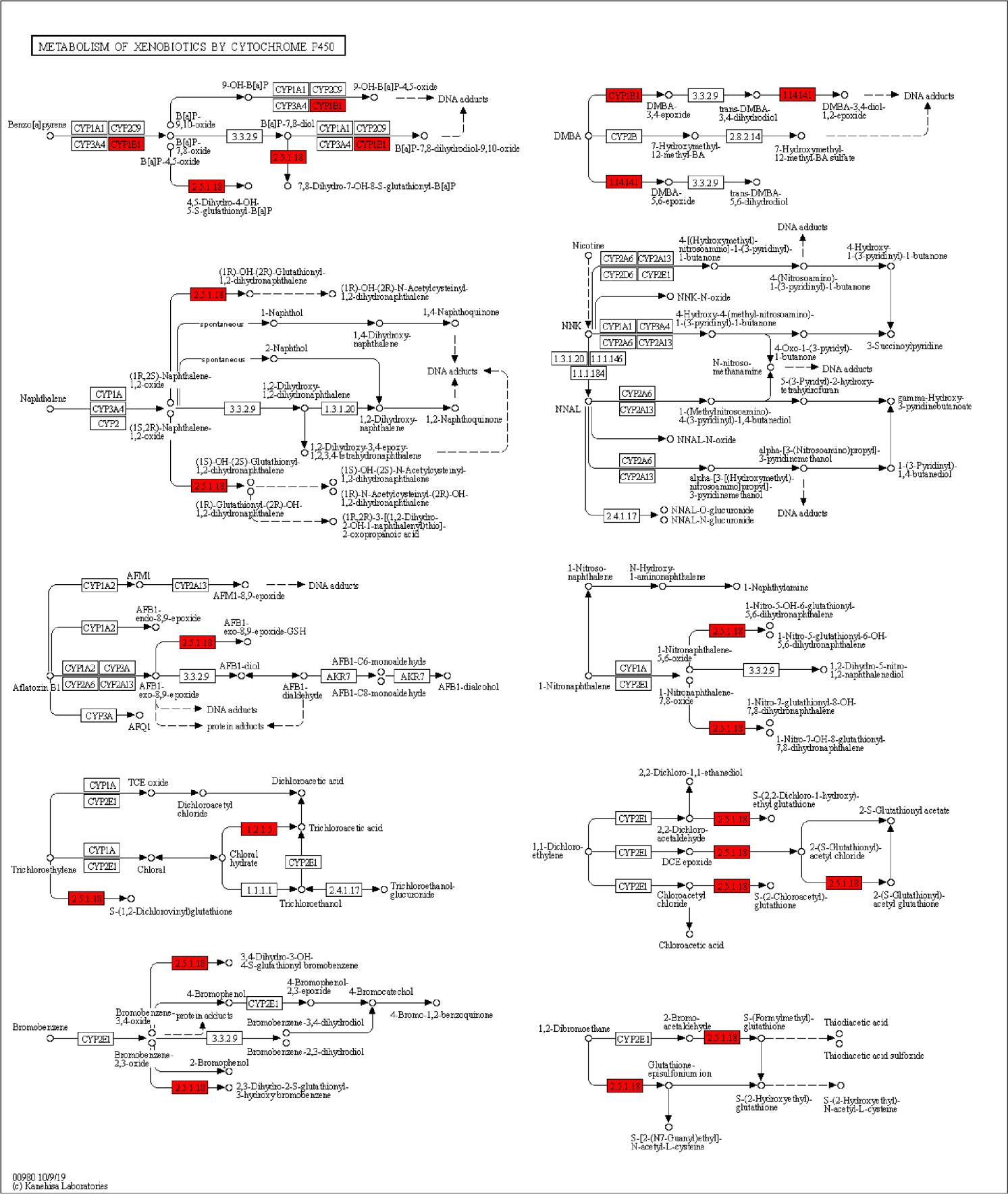


**Supplementary Figure 9.** DEPs that enriched in metabolism of xenobiotics by cytochrome P450 pathway of rHAPLN1 treated RA-FLSs. Targets in red blocks represent up-regulated.


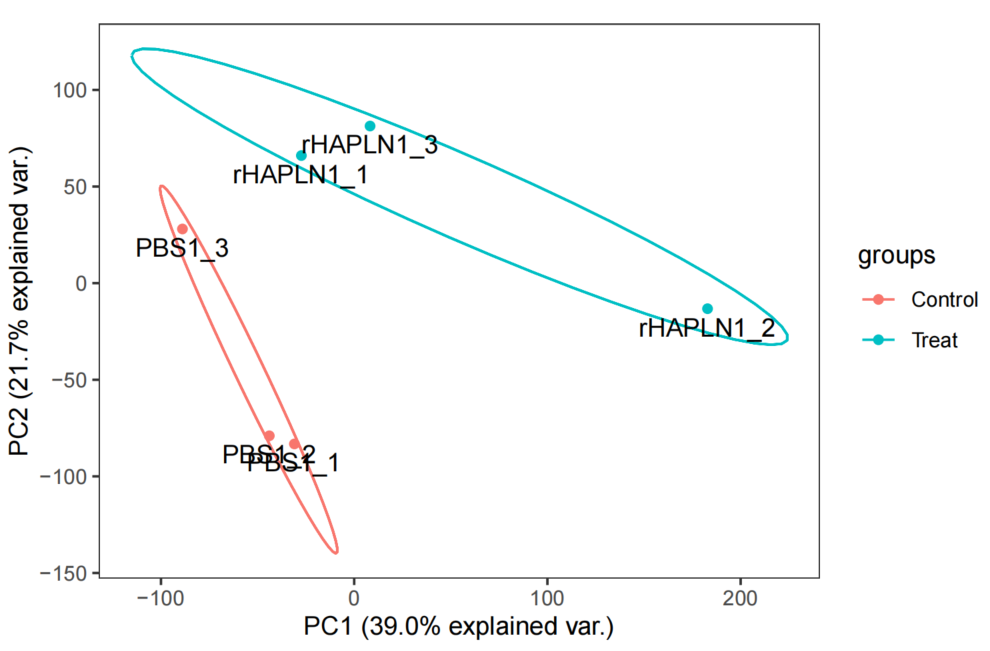


**Supplementary Figure 10.** The principal component analysis (PCA) of all the sample results are shown. Higher aggregation degree between the repeated samples indicates better quantitative repeatability.


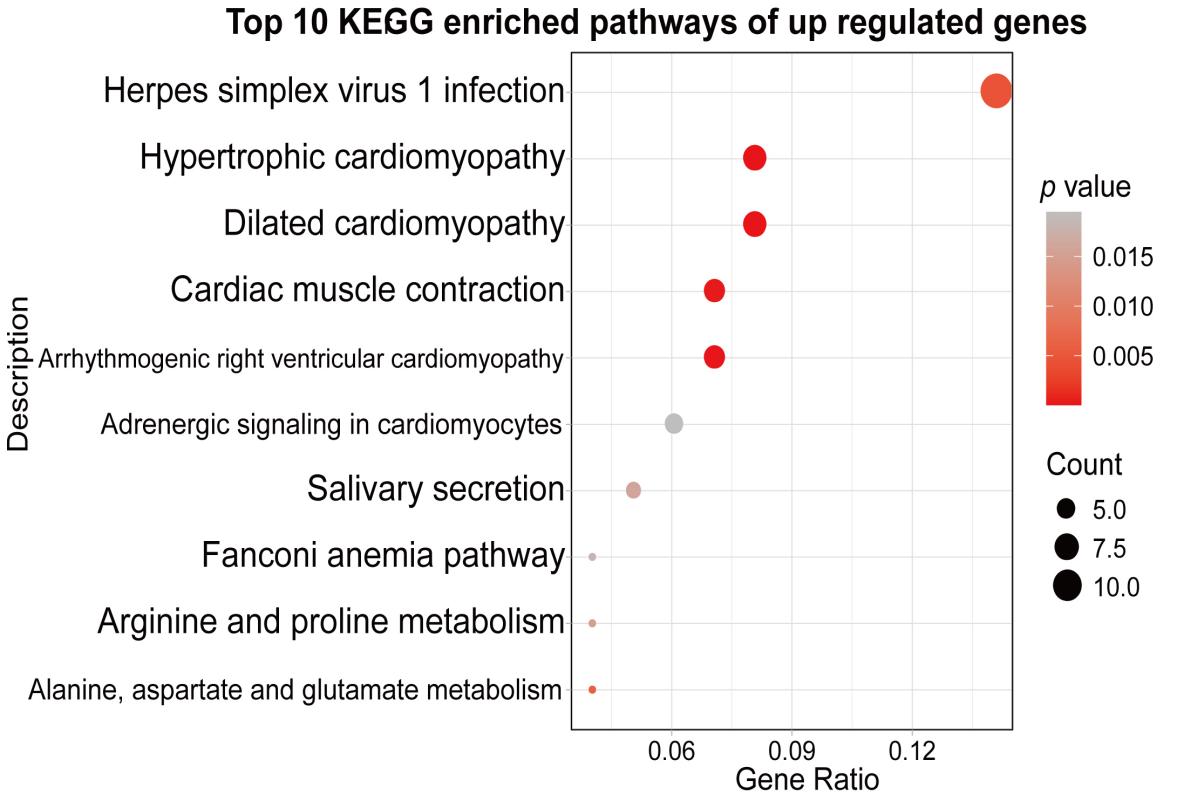


**Supplementary Figure 11.** Top 10 pathways of up-regulated DEGs treated by rHAPLN1.


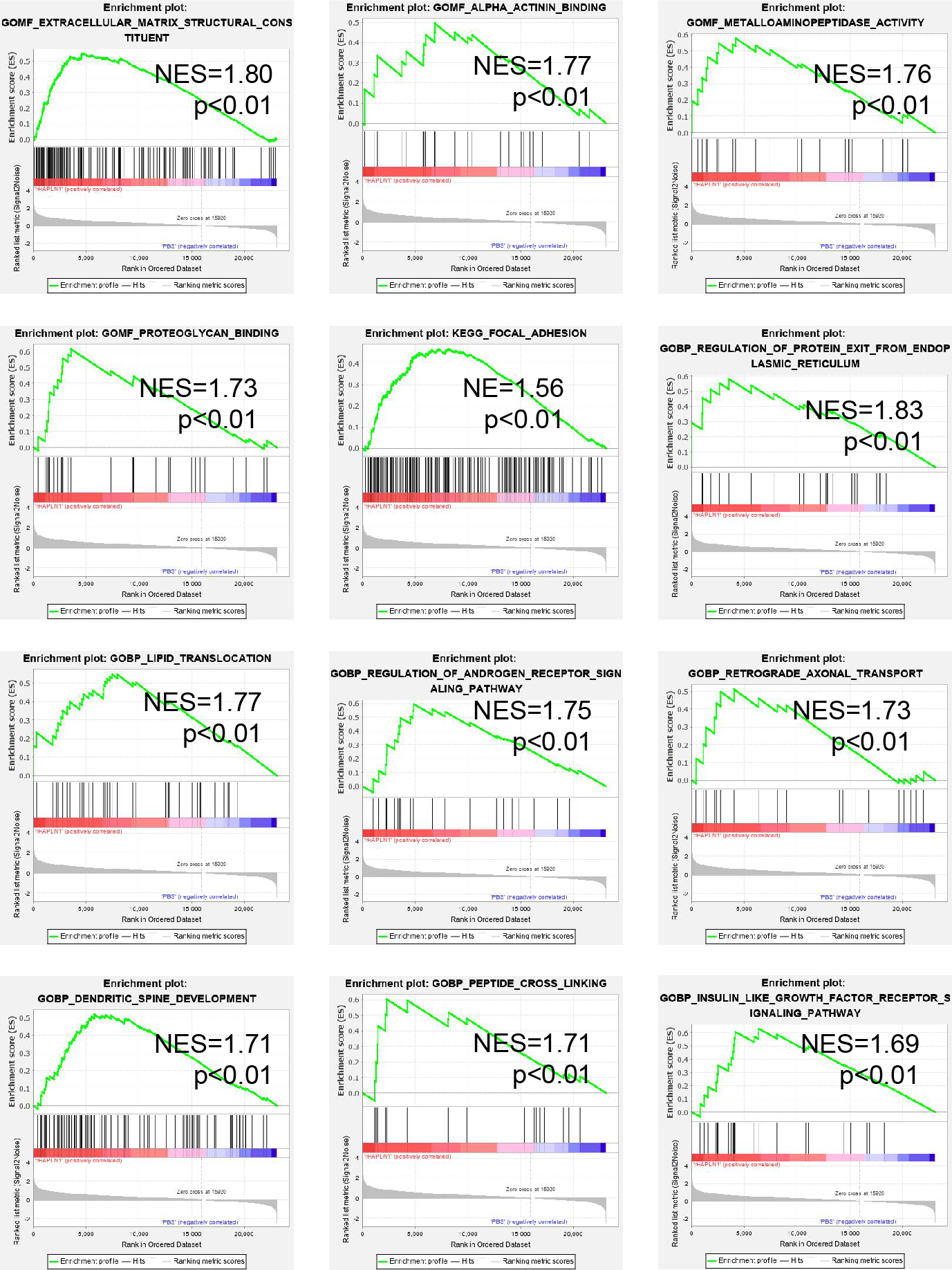


**Supplementary Figure 12.** GSEA analysis shows pathways affected by up-regulated DEGs in rHAPLN1 treated RA-FLSs. Pathways affected include extracellular matrix structural constituent, alpha actin binding, metalloaminopeptidase activity, proteoglycan binding, focal adhesion, regulation of protein exit from endoplasmic reticulum, lipid translocation, regulation of androgen receptor signaling pathway, retrograde axonal transport, dendritic spine development, peptide cross linking, insulin like growth factor receptor signaling pathway etc.
